# Genetic Systems to Investigate Regulation of Oncogenes and Tumour Suppressor Genes in *Drosophila*

**DOI:** 10.3390/cells1041182

**Published:** 2012-12-05

**Authors:** Jue Er Amanda Lee, Nicola J. Cranna, Arjun S. Chahal, Leonie M. Quinn

**Affiliations:** University of Melbourne, Parkville 3010, Melbourne, Australia; E-Mails: jealee@student.unimelb.edu.au (J.E.A.L.); n.cranna@unimelb.edu.au (N.J.C.); aschahal@unimelb.edu.au (A.S.C.)

**Keywords:** *Drosophila*, genetics, cell cycle, development, growth

## Abstract

Animal growth requires coordination of cell growth and cell cycle progression with developmental signaling. Loss of cell cycle control is extremely detrimental, with reduced cycles leading to impaired organ growth and excessive proliferation, potentially resulting in tissue overgrowth and driving tumour initiation. Due to the high level of conservation between the cell cycle machinery of *Drosophila* and humans, the appeal of the fly model continues to be the means with which we can use sophisticated genetics to provide novel insights into mammalian growth and cell cycle control. Over the last decade, there have been major additions to the genetic toolbox to study development in *Drosophila*. Here we discuss some of the approaches available to investigate the potent growth and cell cycle properties of the *Drosophila* counterparts of prominent cancer genes, with a focus on the c-Myc oncoprotein and the tumour suppressor protein FIR (Hfp in flies), which behaves as a transcriptional repressor of c-Myc.

## 1. Introduction

Tight regulation of developmental signaling is essential for controlling the cell growth and division required for proper formation of tissues and organs. Impaired cell cycle progression can be extremely detrimental; reduced cell cycles can lead to small, impaired organs and, conversely, uncontrolled cell proliferation can lead to tissue overgrowth and cancer formation. In *Drosophila*, cell growth and cell cycle progression are regulated by a number of key genes, which have been shown to control cell cycle in an analogous manner in all multicellular organisms. The mammalian *c-Myc* (referred to as Myc from here on) transcription factor and oncogene and its *Drosophila* orthologue, *d-Myc*, are both key regulators of cell growth and division [1,2]. A collection of genetic experiments, transcriptome analyses and genome binding studies in mammals and *Drosophila* have revealed that Myc proteins can bind to the promoters and potentially control the transcription of 10%–15% of all genes [2,3,4,5,6,7] (reviewed in [8]). Thus the regulatory targets of Myc and dMyc include genes from virtually every biochemical and regulatory pathway in the cell, including growth, metabolism, cell cycle progression, differentiation and apoptosis (reviewed in [8,9,10]). 

## 2. Cell Cycle Control is Fundamental to Development and is Disrupted in Cancer: The Myc oncogene and FIR Tumour Suppressor are Conserved between *Drosophila* and Mammals

In addition to regulating a wide range of genes, Myc targets can vary depending on the cell-type and developmental context, a complexity of function which has thwarted attempts to identify a universal transcriptome signature for Myc. In an effort to address these observations, two recent studies ([11,12] and reviewed in [13]) provide a potential explanation for the cell and context dependent variations between transcriptional signatures associated with Myc. Genome-wide ChIP-Seq analysis suggests that rather than driving its own transcriptional program, Myc behaves as a general amplifier of the cells transcriptional state at the time of *Myc* activation. By monitoring co-occupancy of the elongating form of RNA polymerase II and the presence of active chromatin marks, both studies observed that after overexpression, Myc protein is loaded quantitatively onto active promoters to enhance transcription. The observation that *Myc* overexpression does not increase its enrichment within the promoters of silent genes, suggested to the authors that elevation of *Myc* is not sufficient to activate transcription [11,12].

These new models suggest that Myc behaves as an amplifier of existing cellular states, however, *Myc* activation has been largely linked with cell proliferation and elevated levels of Myc are sufficient to drive cell growth and cell cycle progression [1,8,14]. Moreover, the capacity of Myc to drive growth is critical to its oncogenic potential [15]. Indeed, many studies in mammals suggest that Myc is an instructive force, rather than a simple reinforcer of cell fate. In mammals, even physiological levels of Myc can drive quiescent cells to proliferate [8,14]. Extensive studies have shown that like mammalian Myc proteins, dMyc is not only essential but is also sufficient for accumulation of cellular mass or cell growth [1,2,16]. In *Drosophila*, *dMyc* regulates cell and organismal size; hypomorphic mutants of *dMyc* are small due to reduced cell growth [1], while null *dMyc* mutations lead to larval lethality due to a growth arrest [16]. Conversely, overexpression of *dMyc* produces larger flies [1,16]. A pertinent question then remains: how does Myc achieve this effect on growth?

Transcription of ribosomal DNA (rDNA) is required to produce functional ribosomes, which is one of the most fundamental rate-limiting steps for growth and DNA synthesis. *Myc* and *dMyc* are both key regulators of rDNA transcription, ribosome biogenesis and cell growth [1,2]. The *Myc* oncogene regulates cell growth via three RNA polymerases; RNA polymerase I, II and III [17]. *Myc* regulates a large number of RNA polymerase II-transcribed genes, many of which encode ribosomal proteins and translation factors [6]. *Myc* is also very efficient at activating transcription via RNA polymerase I (Pol I) [2,7] and III (Pol III) [18] to drive rDNA transcription, ribosome biogenesis and protein translation. *Drosophila* microrarrays revealed upregulation of Pol I transcripts in *dMyc* overexpressing cells, and an associated increase in rDNA transcription, ribosome biogenesis and cell growth [2]. The capacity to drive production of rRNA (ribosomal RNA) is central to mammalian Myc's powerful cell growth effects and oncogenic ability [15]. Small changes, either up or down, in Myc protein levels will modify growth and potentially result in cancer initiation and/or progression, emphasizing the requirement for extremely tight control of *Myc* expression [19].

**Figure 1:**
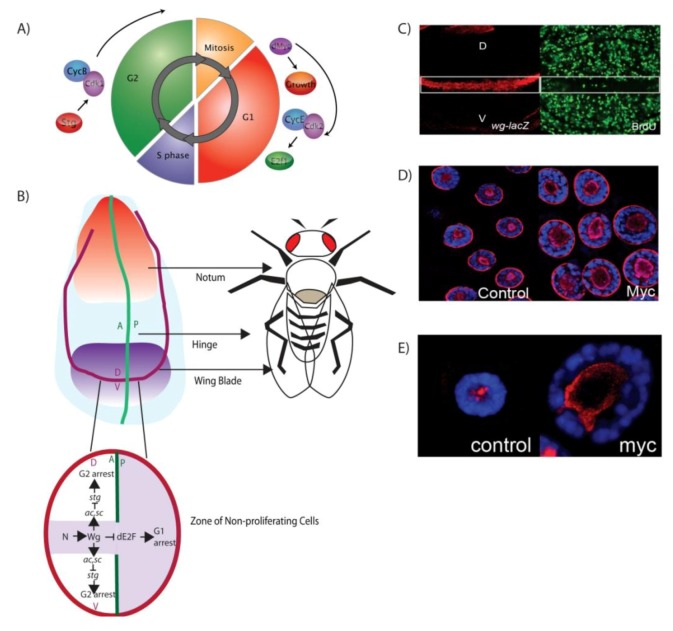
(**A**) dMyc drives ribosome biogenesis and cell growth and promotes S phase, CycE/Cdk2 triggers S-phase by activating E2F1. Stg/Cdc25 promotes G2-M progression by activating the Cdk1/Cyclin B complex (**B**) *Drosophila* wing imaginal disc. The red and blue region develops to form the notum and hinge, while the purple “pouch” region forms the wing blade. The green line marks the anterior-posterior (A/P) boundary while the red line defines the dorsal-ventral (D/V) boundary. Across the D/V boundary, the “zone of non-proliferating cells” (ZNC), is defined by Notch (N) triggering activation of Wingless (Wg). In the anterior ZNC, Wg induces G2 arrest via inhibition of Stg and in the posterior Wg induces G1 arrest via repression of dE2F. (**C**) Wg protein (red) is expressed along D/V boundary and correlates with reduce S phases detected with BrdU (Green). (**D**) dMyc drives ribosome biogenesis and cell growth in *Drosophila* salivary gland cells. Increased nucleolar size is shown with the fibrillarin (red) and (**E**) *In situ* hybridization shows increased 5'ETS (red) in Myc over expressing cells compared to the control.

In addition to the ability to drive growth in *Drosophila* and mammals, the Myc protein is also required to couple growth with cell cycle progression. Like *Myc*, early in the G1 phase of the cell cycle, *dMyc* activates the genes required for DNA replication and progression through S phase [1,2,4,16,20,21,22]. *Myc* and *dMyc* activate the G1 to S-phase Cyclin/Cyclin-dependent-kinase complexes, CycE/Cdk2 and CycD/Cdk4, in order to trigger DNA replication and S-phase progression [23,24,25] (Figure 1A). As the loss of cell cycle control resulting from misregulation of *Myc* is associated with cancer [26,27], *Myc* must be tightly regulated during normal development [14,19,28,29]. The importance of maintaining a tight control of *Myc* levels is reflected by the multiple types of regulation observed for *Myc* and *dMyc*, including transcriptional initiation, RNA synthesis, translation and protein stability [8,30]. Therefore, the upstream signaling pathways and transcriptional, translational and proteolytic mechanisms that regulate *Myc* are of critical importance. In addition, the levels of *Myc* expression need to be responsive to growth and developmental signals to allow organ and tissue growth in response to nutrients and other external cues [1,20,31,32,33]. 

In mammals, one mechanism proposed for tight regulation of *Myc* levels and a quick response of *Myc* transcription to growth signals *in vitro* involves the presence of a paused, but transcriptionally engaged, RNA polymerase II (Pol II) within the *Myc* promoter [9,29,34,35]. Escape of Pol II from the promoter allows transcript elongation. Biochemical evidence has shown the movement of Pol II and elongation of the *Myc* transcript is dependent on the Transcription Factor IIH (TFIIH) complex and its DNA helicase subunit, XPB. Interactions with TFIIH/XPB helicase and two DNA structure-sensitive regulatory proteins called FUSE Binding Protein (FBP) and FBP Interacting Repressor (FIR) control the Pol II complex movement within the promoter of the *Myc* gene [35,36]. In this system, FBP and FIR act as dominant regulators of *Myc*: FBP is a potent activator of *Myc*, while FIR is required for repression of *Myc* transcription. FBP and FIR interact antagonistically and are essential for the transcriptional regulation of *Myc* transcription, and their actions are mediated by the XPB DNA helicase [9,29,34,35,37]. Consistent with a role for FIR in transcriptional repression of *Myc*, loss-of-function FIR mutations are associated with increased *Myc* mRNA levels in primary human colorectal cancer [38]. Mutations of *XPB* [35,36] and activation of FBP [39] have also been implicated in human cancer. 

Evidence suggests *Drosophila* Hfp is the functional homolog of FIR, being essential for repression of *dMyc* transcription and, furthermore, cell growth *in vivo* [31,40]. ChIP experiments have revealed enrichment of Hfp proximal to the *dMyc* transcriptional start site (TSS). In addition, loss of Hfp results in enhanced cell growth, which depends on the presence of dMyc. Furthermore, Hfp physically and genetically interacts with the XPB helicase component of the TFIIH transcription factor complex, Hay, which is required for normal levels of *dMyc* expression, cell growth and cell cycle progression. Below we review how the *Drosophila* model has been used to increase our understanding of how *Myc* expression is correctly patterned and modulated in multicellular organisms *in vivo*, particularly in response to the Hfp transcriptional repressor.

## 3. Drosophila Models for Understanding the Genetics of Cell Cycle Control

### 3.1. Larval Imaginal Discs: Models for Connecting Developmental Signals to Organ Growth

*Drosophila* has been used extensively as a model organism to understand the link between developmental signaling pathways and cell cycle progression [41,42]. A major benefit of this model organism is its short lifecycle. Within 10 days of egg deposition, the *Drosophila* larvae proceeds through the various stages (1st, 2nd, 3rd instar larvae and pupae) to eclosure as an adult fly. Each *Drosophila* larvae contains imaginal discs which are the precursors to many adult organs. Imaginal discs develop from invaginations of the embryonic epithelium into head structures (mouth parts, eyes and antenna), appendages (legs and wings) and genitalia. At the 1st instar stage, imaginal discs are bags of around 10–50 undifferentiated cells, which undergo massive growth and proliferation to comprise up to 100,000 cells by the end of the third larval instar. Differentiation starts at the end of the 3rd larval instar and is complete by the end of pupariation, when all adult structures such as the wings, legs and eyes have developed [43]. The 3rd larval instar is therefore a critical stage of *Drosophila* development, containing the major growth and proliferation of all tissues required to form the adult fly. 

The wing imaginal disc gives rise to the adult wing blade, hinge and part of the thorax (Figure 1B). Cell cycle patterning in the wing imaginal disc pouch has been particularly well characterized [1,41,44,45,46]. For instance, clear connections have been made between developmental signals and the cell cycle delay that occurs late in the 3rd larval instar along the D/V boundary, which is required for differentiation of the margin of the wing blade (Figure 1B) [1,30,41,42,44,45]. A major signaling molecule required for wing disc morphogenesis is the Wingless (Wg) protein, the founding member of the Wnt family of secreted morphogens [47,48]. Wg is secreted across the D/V boundary of the wing pouch during the 3rd larval instar and is required for inhibition of cell cycle and formation of the zone of non-proliferative cells (ZNC) (Figure 1B) [44,46]. This zone gives rise to the adult wing margin and is characterized by reduced S phases as measured by incorporation of bromodeoxyuridine (BrdU) (Figure 1C). The posterior portion of the ZNC is composed entirely of G1 cells, but the anterior portion of the ZNC can be divided into three subdomains (Figure 1B). The central domain, where cells express Wg, is comprised of G1 cells and is flanked by the dorsal and ventral subdomains, where proneural transcription factors Achaete (Ac) and Scute (Sc) are expressed to delay these cells in G2 [44]. Wg induces G2 arrest via Ac and Sc, which act to repress the mitotic factor Stg/Cdc25 in the dorsal and ventral domains of the anterior compartment to result in cell cycle exit and differentiation [44]. In the posterior and central domain of the anterior compartment, Wg inactivates dE2F and results in G1 arrest in preparation for differentiation into the vein and intervein components of the adult wing during the pupal stage [44] (Figure 1B).

In the cell division cycles of the wing imaginal disc pouch, DNA synthesis is coupled with cell division; cells grow in G1, initiate DNA replication and enter S phase, which is separated from mitosis by G2. In these cells G1 progression is stimulated by growth factors, which trigger cell growth and activate the G1-S cell cycle machinery. The inhibition of Rbf, a member of the *Drosophila* Retinoblastoma family [22,49,50], results in release of E2F1 from the inhibitory complex with Rbf, which permits upregulation of E2F1 dependent S phase genes [51]. Imaginal disc cells maintain their size via the tight coupling of cell growth and cell cycle progression. S phase and G2-M can be coupled as CycE and Stg, the rate limiting factors for S-phase and mitosis, respectively, can both be activated by the *Drosophila* orthologue of human E2F1 protein, dE2F1, which thereby coordinates progression from S-phase into mitosis to maintain organ size [52].

### 3.2. The Drosophila Salivary Gland: Models for Understanding Cell Growth Control

In the mitotically dividing imaginal tissues discussed above, cell growth and cell cycle progression are tightly coordinated to allow cells to maintain a consistent size, which can mask potential effects on cell growth. In contrast, the salivary gland and many other tissues in the *Drosophila* larvae undergo endoreplication, *i.e.*, cell growth and DNA replication/S phase occur in the absence of cell division to result in large polyploid cells [53]. Indeed, the salivary gland has been used as a tissue to study the effect of dMyc on cell growth. In these endoreplicating cells, *dMyc* is both necessary and sufficient for rDNA synthesis and growth [2,16]. Salivary gland cells fail to grow and endoreplicate in *dMyc* mutants, while overexpression of *dMyc* dramatically increases cell growth (nucleolar size) and nuclear DNA content (DNA replication) [2,16]. 

In all eukaryotes, the clusters of rRNA genes are organized in the nucleolus, where rRNA transcription, processing and ribosome assembly occur [54]. Thus, nucleolar size is largely proportional to the level of rRNA transcription and provides an indirect measure of ribosomal gene transcription. As predicted, based on previous studies [2] overexpression of *dMyc* in the salivary gland results in increased nucleolar size, measured using an antibody to the nucleolar protein Fibrillarin in 3rd instar salivary glands (Figure 1D). In addition, altered rDNA transcription can be measured directly in salivary glands by monitoring abundance of rRNA. As transcription of the rDNA repeat sequence by RNA Pol I begins within the 5' external transcribed spacer (ETS) region, fluorescent *in situ* hybridisation (FISH) using a DIG-labelled riboprobe for the 5'ETS can be used as a direct read out of rRNA abundance (Figure 1E). Consistent with the increase in the size of the nucleolus detected with fibrillarin, dMyc overexpression results in more 5'ETS, as expected based on the ability of dMyc to increase ribosomal gene transcription [2].

### 3.3. The Drosophila hemopoietic System; a Model for Mammalian Blood Development and Disease: Key Oncogenic Signaling Pathways Drive Overproliferation of Hemocytes

Disruptions to normal hematopoietic function have been implicated in numerous forms of leukemia, anemia and other blood disorders. The first link between dysregulation of the *Myc* oncogene and human disease was in Burkitts lymphoma, where the *Myc* gene is translocated to the immunoglobulin heavy chain locus, which results in amplified *Myc* expression (reviewed in [55,56,57]). One of the many problems faced by medical researchers attempting to dissect the underlying cause of hemopoietic disease is the complex genetic control and functional redundancies acquired as a result of gene duplication and evolution in mammals. Morphologically, development of the *Drosophila* and mammalian blood lineages is considerably diverged. However, molecular genetic studies have revealed that many key hemopoietic signaling pathways and transcription factors are conserved between systems [58,59,60,61,62,63]. 

**Figure 2:**
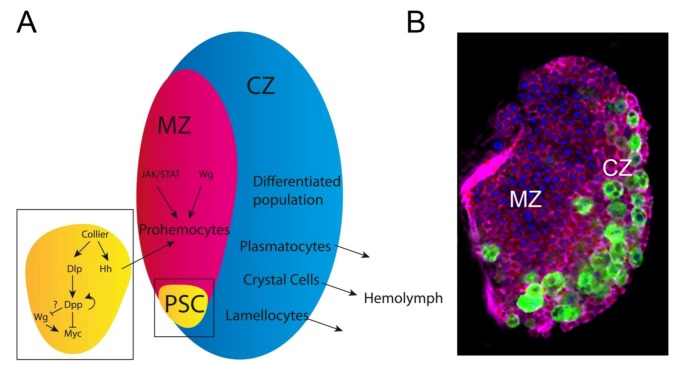
(**A**) Developmental signaling in the Lymph Gland. The primary lobe is divided into three subsections: the medulla (MZ, composed of prohemocytes or hematopoietic precursor cells), the cortex (CZ, predominantly comprising plasmatocytes and crystal cells that migrate into the hemolymph) and the PSC, which signals to the MZ to maintain blood cell homeostasis. (**B**) A primary lobe of a third instar larval stained for DNA (blue) and actin (pink), showing crystal cells marked by *lozenge-GAL4/UAS-GFP*.

In *Drosophila*, the blood producing lymph gland (LG) is specified prior to the blastoderm stage of embryogenesis [64]. The LG initially consists of 2 lobes, known as the primary lobes, which first appear late in the embryonic stages. Expression of *collier*, the *Drosophila* orthologue of the vertebrate transcription factor Early B Cell Factor, is one of the earliest markers of LG precursors [65]. *Collier* expression becomes increasingly restricted until it is only expressed in the most posterior 2–3 cells of the approximately 20–25 cell LG, the future Posterior Signaling Center (PSC, see Figure 2). The LG persists as the larval hematopoietic organ until the onset of metamorphosis, at which point it disintegrates and releases its contents into the hemolymph. There is no evidence of a hematopoietic organ in the adult *Drosophila*.

The fully mature 3rd instar larval LG (Figure 2) consists of a pair of primary lobes and 2–7 pairs of secondary lobes which develop during successive rounds of proliferation during the early 3rd instar. The primary lobe is divided into three subsections: the medulla (MZ, composed of prohemocytes or hematopoietic precursor cells), the cortex (CZ, comprising differentiating and mature cells) and the PSC, which is responsible for maintaining larval blood cell homeostasis [66,67,68]. Under normal conditions, two cell types are found in the LG: (1) plasmatocytes and (2) crystal cells [64]. Plasmatocytes make up the vast majority of the hemocyte population (~95%) and function to phagocytose bacterial or fungal infections along with removal of excess cells during development [69]. 

A number of key signals have been implicated in both the mammalian and *Drosophila* hematopoietic maintenance as well as lineage specification. Dysregulation of these pathways can lead to loss of hemocyte progenitors and/or differentiation blocks, and can result in LG overgrowth in *Drosophila* and malignancy in humans. Ras was amongst the first human oncogenes identified and belongs to the family of genes encoding GTP-binding proteins. This family of genes is implicated in regulating cell growth, proliferation, and differentiation and is dysregulated in approximately 30% of human cancers [70,71]. In *Drosophila* expression of the activated/oncogenic form of Ras (Ras^V12^) specifically in the hemocytes results in a 40-fold increase in hemocyte number [59]. The overproliferation can be suppressed by reducing Cdk2/cyclin E, suggesting the Ras-induced increase in hemocytes is a result of increased E2F activity [59]. These changes in cell proliferation may depend on Myc, as the Ras pathway signals through the MAP kinase pathway to both increase Myc/dMyc protein levels [72,73,74]. Consistent with this, in the *Drosophila* wing imaginal disc, Ras activity results in increased dMyc protein accumulation and accelerated cell cycle progression [75]. The ability to generate leukemia-like phenotypes with well-established oncogenes lends weight to the idea that *Drosophila* may be a useful model for human blood disorders.

Further to this, the pathways implicated in PSC signaling and stem cell behavior also regulate activity of the mammalian HSC niche. The TGF-β super family member BMP4 regulates the mouse HSC niche [76] and in *Drosophila* hematopoiesis the orthologous BMP protein, Dpp, is necessary for maintaining PSC size and, as a result, larval blood homeostasis [63]. Similarly, Wg, and the BMPF1 ortholog, Dlp, are modulated by Dpp signaling and are required for hematopoiesis (Figure 2A) [63]. Thus, many parallels exist between mammalian and *Drosophila* hematopoietic niches, and given the role of Myc in controlling development of the HSC lineage [77,78] and leukemia [55,56,57] it will be of interest to investigate potential roles for dMyc in *Drosophila* hematopoiesis in the future.

## 4. Genetic Tools for Manipulating Gene Expression in Drosophila

**Figure 3:**
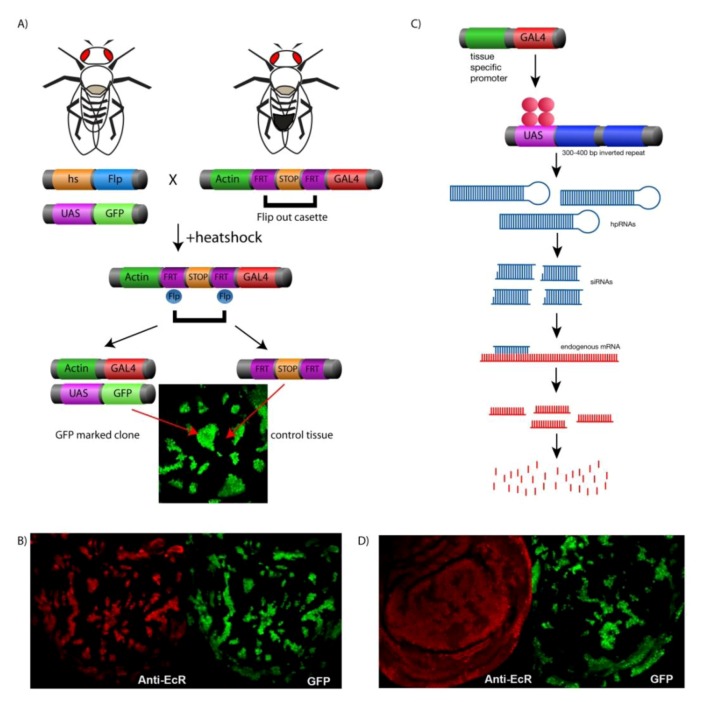
(**A**) The *hsflp*, *Actin*<*Gal4* system. Heat shock induces FLP expression and recombination removes the intervening stop sequence from the construct. The actin promoter drives GAL4 for *UAS*-transgene(s) expression in clones, including *UAS*-*GFP* to mark the clone (**B**) Wing imaginal discs from wandering third instar larvae containing "Flip out" clones marked with GFP (Green), overexpressing the ecdysone receptor gene (EcRB2) detected by the ecdysone receptor antibody (red). (**C**) "Flip out" clones expressing EcR RNAi marked with GFP showed ablation of ecdysone by the reduction of ecdysone antibody (red).

**Figure 4:**
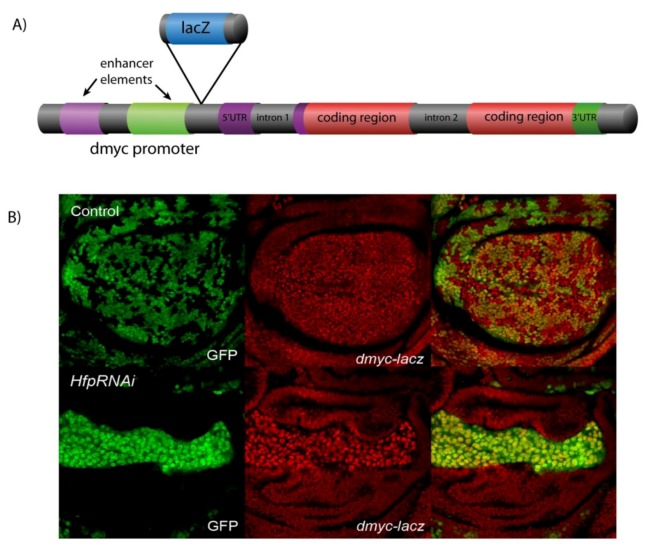
(**A**) Schematic diagram of a *dMyc-lacZ* enhancer trap. (**B**) *dMyc* enhancer trap activity detected with β-gal (red) for wing imaginal disc containing control or Hfp RNAi GFP positive clones (green).

The *UAS-GAL4* system, derived from the yeast *S. cereviase*, has provided an invaluable genetic tool for studying the manipulation of gene expression during *Drosophila* development [79]. With a diverse variety of GAL4 drivers, temporal and tissue specific overexpression of a given *UAS*-transgene can be achieved in a specific tissue of interest. For example, cell cycle biologists combined the *UAS-GAL4* system with the *FLP/FRT* system to generate the Actin<*CD2*<*GAL4* “flip out” system in order to monitor cell growth and division in patches of tissue or “clones” over time (Figure 3A [80,81]). Gene expression is controlled by heatshock induced expression of the Flip recombinase (*Flp*) [82]. *Flp* will recognise the *FRT* sites in the Act<*CD2<GAL4* cassette. The “flipping out” of the interruption cassette results in: (1) CD2 protein expression in the neighboring control clone and (2) the Actin promoter driving *GAL4* in the clone, which can be positively marked with co-expression of a fluorescent transgene e.g. *UAS*-*GFP* or *RFP* with the *UAS*-transgene of interest (Figure 3A,B) [80,81,83]. This system is ideal for studying effects of manipulating gene expression on cell growth, proliferation, and signaling pathways in comparison to surrounding normal tissue, which is essential for understanding tumorigenesis. For gene knockdown, GAL4 drivers can be used for expression of a *UAS*-transgene for an inverted repeat targeting the gene of interest [84]. Extensive *UAS*-RNAi collections have been developed and made publicly available and, as these cover most (97%) of the *Drosophila* genome (e.g. Vienna *Drosophila* resource centre [85]), they have provided an invaluable resource for conducting non-biased genetic screens to elucidate novel signaling mechanisms and gene function (Figure 3C,D). For example, “flip-out” clones generated using a hairpin targeting Hfp, were used to demonstrate that loss of Hfp results is increased growth compared with the surrounding control cells [40]. Moreover, using the *dmyc*-*lacZ* enhancer trap described below, we have demonstrated that Hfp is required for repressing *dmyc* promoter activity (Figure 4, [40]).

In mammalian systems, *in vitro* transcription assays (e.g., luciferase assays) are a standard method for monitoring effects of a particular factor on promoter/enhancer activity. However, if we are to draw connections between the signaling environment and changes to the expression of cell cycle genes, it is essential to investigate transcription from endogenous promoters *in vivo*. In *Drosophila*, promoter and enhancer trap activity can be measured using reporter elements inserted into endogenous promoters in order to determine factors capable of modulating transcription of a given gene. Furthermore, random P-element mutagenesis screens generated extensive enhancer trap collections, where each line contains the insertion of a visible reporter (e.g., LacZ or GFP) for monitoring gene activity within a particular region of the genome [86,87]. As these enhancer traps often land in regions of active chromatin, such as gene promoters, they can often be used to measure endogenous promoter or enhancer activity, as an indicator of transcriptional activation of the gene located downstream of the insertion [88]. 

For example, a random *P-lacZ* insertional mutagenesis screen for the X-chromosome resulted in many hits in the *dMyc* promoter [89], providing potential enhancer traps to identify changes in *dMyc* promoter activity. Analysis of these *lacZ* insertions revealed that insert [*P{lacW}l(1)G0354* [89]], located just prior to the 5'UTR in the *dMyc* promoter, in a region that is responsive to wing enhancers of *dMyc* expression (Figure 4A). By using the ®-gal antibody, this *dMyc-lacZ* line can, therefore, be used to monitor changes to *dMyc* promoter activity in wing imaginal discs [40,42]. In particular, *UAS-hfp* RNAi “flip-out” clones in the *dMyc*-*lacZ* enhancer trap background, revealed that Hfp is required for repression of *dMyc* promoter activity (Figure 4B and [40]). The enhancer trap appears to reflect changes in *dMyc* transcription, as mRNA abundance is significantly increased in Hfp loss of function imaginal tissues [40] and can provide an *in vivo* read out for potential enhancers and suppressors of *dMyc* transcription.

## 5. Conclusion

As detailed above, Myc is a potent mitogen, but despite the large number of transcriptional targets and the associated oncogenic potential of *Myc* dysregulation, how *Myc* is regulated at the transcriptional level is largely unknown. In *Drosophila* using the genetic systems described above, we have demonstrated that repression of *dMyc* promoter activity and cell growth requires Hfp function (Figure 4B and [40]). Furthermore, the increased *dMyc*-*lacZ* reporter activity, compared to surrounding wild type cells (Figure 4B) was associated with significantly increased *dMyc* mRNA levels [40]. Thus like its mammalian counterpart FIR, Hfp behaves as a tumor suppressor to repress *dMyc*, which suggests that the mechanism proposed for transcriptional repression of *c*-*Myc* by FIR is conserved in *Drosophila*. These data suggest that the loss-of-function FIR mutants described in colorectal cancer may be sufficient to increase *Myc* expression, which would be predicted to lead to cancer initiation and progression. 
